# In Vitro and Clinical Evaluation of Cannabigerol (CBG) Produced via Yeast Biosynthesis: A Cannabinoid with a Broad Range of Anti-Inflammatory and Skin Health-Boosting Properties

**DOI:** 10.3390/molecules27020491

**Published:** 2022-01-13

**Authors:** Eduardo Perez, Jose R. Fernandez, Corey Fitzgerald, Karl Rouzard, Masanori Tamura, Christopher Savile

**Affiliations:** 1 Research and Development Department, Signum Biosciences, 11 Deer Park Drive Suite 202, Monmouth Junction, NJ 08852, USA; eperez@signumbio.com (E.P.); jfernandez@signumbio.com (J.R.F.); cwebb@signumbio.com (C.F.); krouzard@signumbio.com (K.R.); mtamura@signumbio.com (M.T.); 2 Willow Biosciences, Inc., 1201-5th St. SW, Suite 202, Calgary, AB T2L 1Y8, Canada

**Keywords:** fermentation, anti-aging, antioxidant, cannabidiol, cannabigerol, cannabis

## Abstract

Cannabigerol (CBG) is a minor non-psychoactive cannabinoid present in *Cannabis sativa* L. (*C. sativa*) at low levels (<1% per dry weight) that serves as the direct precursor to both cannabidiol (CBD) and tetrahydrocannabinol (THC). Consequently, efforts to extract and purify CBG from *C. sativa* is both challenging and expensive. However, utilizing a novel yeast fermentation technology platform, minor cannabinoids such as CBG can be produced in a more sustainable, cost-effective, and timely process as compared to plant-based production. While CBD has been studied extensively, demonstrating several beneficial skin properties, there are a paucity of studies characterizing the activity of CBG in human skin. Therefore, our aim was to characterize and compare the *in vitro* activity profile of non-psychoactive CBG and CBD in skin and be the first group to test CBG clinically on human skin. Gene microarray analysis conducted using 3D human skin equivalents demonstrates that CBG regulates more genes than CBD, including several key skin targets. Human dermal fibroblasts (HDFs) and normal human epidermal keratinocytes (NHEKs) were exposed in culture to pro-inflammatory inducers to trigger cytokine production and oxidative stress. Results demonstrate that CBG and CBD reduce reactive oxygen species levels in HDFs better than vitamin C. Moreover, CBG inhibits pro-inflammatory cytokine (Interleukin-1β, -6, -8, tumor necrosis factor α) release from several inflammatory inducers, such as ultraviolet A (UVA), ultraviolet B (UVB), chemical, *C. acnes*, and in several instances does so more potently than CBD. A 20-subject vehicle-controlled clinical study was performed with 0.1% CBG serum and placebo applied topically for 2 weeks after sodium lauryl sulfate (SLS)-induced irritation. CBG serum showed statistically significant improvement above placebo for transepidermal water loss (TEWL) and reduction in the appearance of redness. Altogether, CBG’s broad range of *in vitro* and clinical skin health-promoting activities demonstrates its strong potential as a safe, effective ingredient for topical use and suggests there are areas where it may be more effective than CBD.

## 1. Introduction

The use of *Cannabis sativa* L. (*C. sativa*) for medicinal purposes such as ameliorating inflammation, pain, sleep, and neurological disorders dates back several thousands of years to its use in ancient China [1]. Since that time, over 100 cannabinoids have been identified in *C. sativa* and it is this group of bioactive molecules that appear to be responsible for conferring the plants many health benefits. More recently, evidence for the use of *C. sativa* extracts and its individual cannabinoids for skin health and the treatment of dermatological conditions has emerged [2]. The most well-known and studied of the cannabinoids is cannabidiol (CBD), which has been reported to possess antioxidant, anti-bacterial, anti-acne, and anti-aging properties when studied *in vitro* and topically *in vivo* [3]. This in part has led to a recent surge of CBD use in cosmetics, where it has been marketed as an analgesic, anti-wrinkle, skin brightener, and moisturizer with claims to alleviate pruritus, psoriasis, acne, and eczema [4]. However, despite these wide-ranging claims, the clinical evidence for the use of cannabinoids in skin remains scarce. As of the time of writing, only three clinical observations have been published studying the activity of *C. sativa* extracts and/or CBD topically. The first clinical study was a small, proof-of-concept 11-subject study, where a 3% *C. sativa* extract was found to significantly reduce sebum and erythema better than vehicle, suggesting CBD could be helpful for dry skin and acne [5]. The second study was a single-arm phase I safety study in 20 healthy volunteers, where a patented 5% CBD formulation (synthetically produced CBD-BTX 1503) showed it was well tolerated and reduced acne lesions [6]. However, the subsequent phase II clinical study demonstrated that this 5% CBD formulation failed to beat a placebo for its primary endpoints. The third published clinical evidence for CBD was three case study reports of subjects with epidermolysis bullosa, a rare genetic skin disorder that causes skin fragility and painful blistering. Subjects applying topical CBD oil noted faster wound healing, less blistering and less pain, but the study lacked a vehicle control group [7]. Altogether, this highlights the potential of cannabinoids for dermatological use, but also demonstrates that the research and understanding of cannabinoids for skin application is at an early stage.

With the deregulation of CBD and other *C. sativa*-derived non-psychoactive cannabinoids through the 2018 Farm Bill, there is growing interest in cannabinoid pharmacology. One such cannabinoid starting to garner attention from both researchers and consumer product companies is cannabigerol (CBG), which serves as the direct precursor to CBD and tetrahydrocannabinol (THC) [8]. CBG is a minor non-psychoactive cannabinoid that, like CBD, has been reported to possess anti-inflammatory [9], antioxidant [10] properties and modulate differentiation in keratinocytes [11]. Additionally, CBG has also been reported to increase lipogenesis in sebocytes, which is an activity that has not been shown for CBD [12]. CBG was the first cannabinoid to be isolated from *C. sativa* [2] and is present in low levels (<1% per dry weight), typically mixed with THC and CBD. Consequently, agricultural production and purification of CBG or other minor cannabinoids from *C. sativa* is both challenging and expensive. Chemical synthesis of minor cannabinoids is another option, but its appeal for widespread commercial use has waned as the required multi-step synthesis produces low yields and elevated costs. An emerging new alternative is the utilization of engineered microbial strains to biosynthetically produce cannabinoids [13]. The identification of the enzymes involved in the cannabinoid biosynthetic pathway has opened the door for the reconstruction of the pathway using a suitable heterologous host system [14]. Specifically, utilizing our novel yeast fermentation technology platform, we can produce minor cannabinoids, such as CBG, with higher purity in a more sustainable and cost-effective manner compared to plant-based extraction or chemical synthesis.

Here, we report that biosynthetically produced CBG possesses a broad range of anti-inflammatory, antioxidant, and skin protecting properties to help slow inflammation, aging, and boost skin barrier function. Gene array analysis of CBG and CBD applied topically to a 3D human skin model demonstrates that CBG outperforms CBD, selectively targeting collagen, elastin and other key skin health and hydration genes. Moreover, *in vitro* studies in normal human epidermal keratinocytes (NHEKs) and human dermal fibroblasts (HDFs) show that CBG and CBD both possess strong antioxidant and anti-inflammatory properties, with CBG demonstrating equal if not better activity than CBD. Lastly, we are the first to report the clinical effectiveness of topically applied CBG, demonstrating that a 0.1% CBG serum reduces inflammation, redness, and improves the skin barrier significantly better than a placebo.

## 2. Results

### 2.1. Gene Microarray Analysis of CBG and CBD

**Figure 1:**
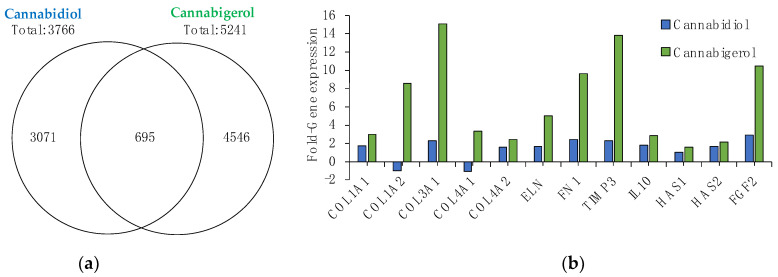
Gene microarray analysis of CBG and CBD. (**a**) Gene differential expression was quantified using Clariom™ S assay microarray system method after 24 h incubation in 3D skin model EpiDerm-FT™. (**b**) Data subset of gene expression related to skin. Data represent mean from *n* = 3 tissues. All genes shown were significantly different (*p* value < 0.05) relative to vehicle-only treated tissues.

To begin to characterize the potential effects of non-psychoactive cannabinoids in skin, CBG and CBD were applied topically to a human 3D skin tissue model (EpiDerm-FT™) and total RNA was isolated after twenty-four hours to perform gene microarray analysis. Results show that 0.5% CBD regulates 3766 genes (1675 upregulated; 2091 down-regulated), while 0.5% CBG modulates over 1400 more genes, totaling 5241 genes (2530 upregulated; 2711 down-regulated) (Figure 1a). CBG and CBD were found to overlap on 695 regulated genes when compared to untreated skin and remarkably, CBG was found to regulate 4546 unique genes, ~50% more than the number of unique genes regulated by CBD (3071 genes) (Figure 1a). In addition to these significant differences in overall gene regulation, CBG and CBD were found to modulate several key genes for skin aging, hydration, and inflammation.

The effects of natural aging and photoaging on the dermis involve negative alterations to the extracellular matrix (ECM) containing collagen. Collagens are essential scaffold proteins that promote skin smoothness and elasticity, but as we age their expression levels (collagen I and type III) decline [15]. Moreover, collagen IV is essential for basement membrane stability and is a key ECM protein [16]. Analysis of these key ECM genes suggests that CBG provides more significant anti-aging benefits to skin than CBD. Results show that CBG significantly boosts Collagen I (COL1A1), III (COL3A1) and IV (COL4A1) by 3-fold, 15-fold, and 3-fold, respectively. Conversely, CBD has a lesser effect on COL1A1 (1.7-fold), COL3A1 (2.3-fold), and lowers COL4A1 expression (−1.1-fold) (Figure 1b). Additionally, while both CBD and CBG promote the expression of elastin (ELN), another ECM protein critical for maintaining skin elasticity [17], fibronectin (FN1), critical for cell growth and wound healing [18] and a tissue inhibitor of metallopeptidase inhibitor 3 (TIMP3), is an important factor for inhibiting UVB-induced inflammation [19], CBG appears to be more effective. Results demonstrate that CBG upregulates ELN, FN1 and TIMP3 by 5-fold, 9.7-fold and 13.8-fold, respectively, as compared to CBD which upregulates the same genes by only 1.7-fold, 2.5-fold and 2.3-fold (Figure 1b). Moreover, CBG was also found to be more effective than CBD in upregulating key hydration gene hyaluronan synthase 2 (HAS2) and fibroblast growth factor 2 (FGF2), which plays an important role in wrinkle reduction through the regeneration and proliferation of skin cells [20,21]. CBG significantly increased HAS2 expression 2.1-fold, while CBD did not reach significance and increased FGF2 expression ~3.5 times better than CBD (10.5-fold vs. 2.9-fold). Lastly, CBG was found to significantly upregulate interleukin-10 (IL-10), a potent anti-inflammatory cytokine [22], by 2.9-fold while CBD had no significant effect (Figure 1b).

### 2.2. CBG and CBD Possess Potent Antioxidant Activity

Several different cannabinoids including CBD and CBG have previously been shown to possess antioxidant activity in cell-free systems [10]. Given our exploration into the potential effectiveness of CBD and CBG in skin, we sought to determine their antioxidant properties by testing their activity as a free radical scavenger in both cell-free and cell-based assays. For cell-free antioxidant capacity, actives were tested for their ability to reduce the free radical DPPH. IC_50_ (inhibitory concentration at 50%) results show that CBG reduces free radical DPPH with an IC_50_ = 502 µM, which was ~2 times more potent than CBD (IC_50_ = 910 µM), but significantly less potent than ascorbic acid (IC_50_ = 5 µM), a commonly used topical antioxidant active (Table 1).

Reactive oxygen species (ROS) production is amplified during inflammatory responses and is a main contributor to accelerating skin aging [23]. Several factors including sun damage, chemicals, and pollution can induce inflammation and intracellular ROS. If intracellular ROS and other reactive species remain unchecked, they can cause oxidative damage to lipids, nucleic acids, and proteins, which ultimately accelerates the natural aging process of our skin [24]. However, the onset of oxidative stress can be delayed by functional antioxidant molecules that can successfully penetrate dermal cells and scavenge damaging oxidative species. To determine if CBG and CBD’s antioxidant activity extended to a cell-based model, we tested their ability to scavenge free radicals in HDFs induced with H_2_O_2_. Our results show that both CBG and CBD significantly inhibit intracellular free radical formation with an IC_50_ = 0.003 nM, which was ~1800-fold more potent than ascorbic acid (IC_50_ = 5.6 nM) (Table 1). Altogether, these data suggest that CBG and CBD not only possess strong antioxidant properties but can also successfully penetrate dermal cells and scavenge damaging ROS inside the cell.

### 2.3. CBG Protects against UVA and UVB-Induced Inflammation and Photoaging

Ultraviolet (UV) light is a common environmental stressor that attacks our skin daily. Longer wavelength UVA penetrates deep into the dermis, while shorter wavelength, higher energy UVB primarily effects the epidermis. Extended exposure to UVB and UVA triggers a cascade of inflammation and production of pro-inflammatory cytokines that play a key role in skin photoaging [25]. Specifically, NHEKs exposed to UVB triggers the release of tumor necrosis factor alpha (TNFα), which has also been linked to dry skin [26]. Our results demonstrate that CBG inhibits TNFα release in a dose-dependent manner (IC_50_ = 14.7 nM), 2-fold more than CBD (IC_50_ = 29.8 nM) (Table 2). Moreover, this strong potency was two orders of magnitude greater than that observed for clobetasol propionate (IC_50_ = 2142 nM), a commonly used topical glucocorticoid (Table 2). UVA also plays a major role in cutaneous photoaging [27] by triggering inflammation and the breakdown of collagen types I, II and III. Specifically, UVA enhances interleukin-6 (IL-6) secretion [28]. To further characterize cannabinoid anti-inflammatory activity, we screened for their ability to reduce UVA-induced IL-6 release in HDFs. CBG significantly inhibited IL-6 production, demonstrating an IC_50_ = 0.3 µM (Table 2) with a 50% maximum inhibition, while CBD tested at 0.3 µM only reached a maximum inhibition of 27% without reaching significance (Table 2). Ascorbic acid tested at its highest non-toxic concentration of 5700 nM did not reduce UVA-induced IL-6 release (Table 2).

### 2.4. CBG Protects against Chemical and C. acnes-Induced Inflammation

**Figure 2:**
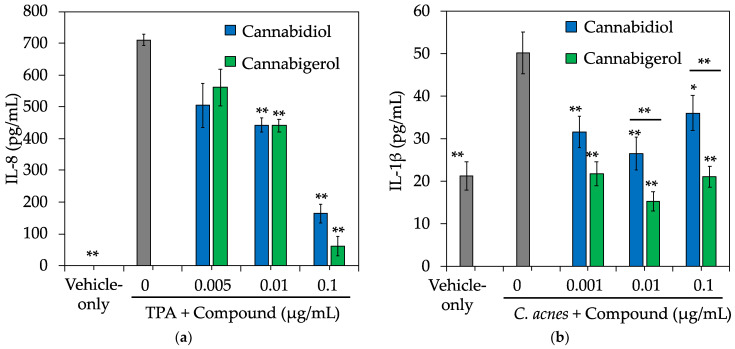
CBG and CBD protect against chemical and bacteria-induced inflammation. Primary NHEKs were cultured in the presence of CBD or CBG for 1 h. Later, cells were co-treated with compounds and 5 ng/mL TPA or 1 × 10^7^ CFU *C. acnes* (ATCC^®^ 6919™) for 24 h. Media supernatants were collected after 24 h and analyzed by ELISA for (**a**) Interleukin-8 (IL-8) or (**b**) Interleukin-1β (IL-1β). Data represent mean ± SE from three independent experiments. * *p* < 0.05; ** *p* ≤ 0.01 relative to inducer + vehicle group.

In addition to UV rays from sunlight, several other environmental stressors can trigger inflammation in skin and if left unprotected, can accelerate the natural skin aging process. Two such culprits are chemicals and bacteria. 12-*O*-Tetradecanoyl-Phorbol-13-Acetate (TPA) is a common chemical irritant used for testing topical *in vivo* anti-inflammatory activity [29]. Furthermore, previous studies demonstrate NHEKs treated with TPA results in the secretion of several pro-inflammatory cytokines [30]. More specifically, NHEKs treated with TPA resulted in a significant induction of interleukin-8 (IL-8) and CBG inhibited its release in a dose-dependent manner with an IC_50_ = 48 nM, which was ~5-fold more potent than CBD (IC_50_ = 233 nM) (Figure 2a). Moreover, CBG was found to be ~40-fold more potent than clobetasol propionate in this assay (IC_50_ = 2100 nM) (data not shown).

Toll-like receptors (TLRs) are critical in regulating the innate immune response to pathogens in skin, and TLR-2 signaling has been shown to play a key role in the pathogenesis of several dermatological conditions including acne [31]. While the pathogenesis of acne has yet to be fully understood, the Gram-positive bacterium *Cutibacterium acnes* (*C. acnes*) has been identified as a key player for this inflammatory skin condition [32] and activation of TLR-2 by *C. acnes* has been reported to trigger inflammatory cytokine responses in acne [33]. Moreover, TLR-2 expression is increased in acne lesions [34]. Our results show that CBG and CBD dose-dependently reduces *C. acnes*-induced interleukin-1beta (IL-1β) release in NHEKs with IC_50_ = 0.0003 nM (Figure 2b), which was several orders of magnitude more potent than the anti-inflammatory activity observed for control glucocorticoid dexamethasone (IC_50_ > 2500 nM) (data not shown).

### 2.5. CBG Improves Skin Barrier Function and Reduces the Appearance of Redness in Human Subjects

**Figure 3:**
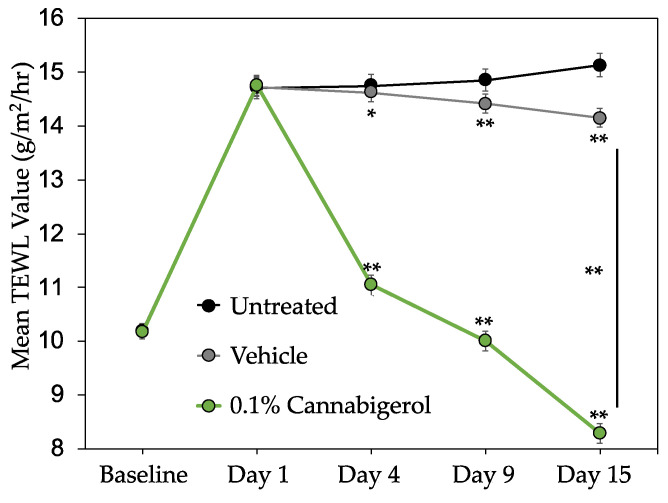
Clinical study utilizing 0.1% CBG in serum formulation shows improvement in hydration levels over untreated and vehicle-treated skin. Transepidermal water loss (TEWL) was measured with a Tewameter^®^ TM300 instrument with lower readings demonstrating an increase in hydration and skin barrier function. Data (*n* = 20) represent mean ± SEM. * *p* < 0.05; ** *p* ≤ 0.01 indicates a statistically significant difference compared to baseline reading.

**Figure 4:**
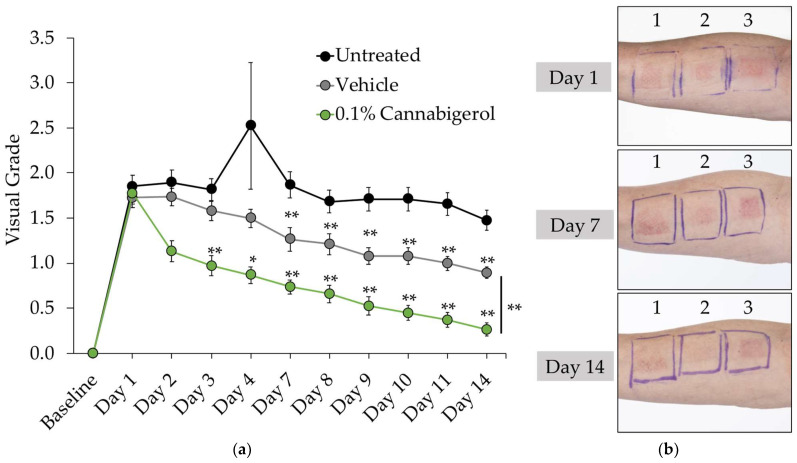
Skin irritancy was improved by 0.1% CBG in serum formulation as measured by visual grading. (**a**) Visual grade data represent mean ± SEM from 20 participants from each group. * *p* < 0.05; ** *p* ≤ 0.01 indicates a statistically significant difference compared to untreated group. (**b**) Photographs of a representative subject on day 1, 7, and 14 of the study where patch 1 = Untreated; 2 = 0.1% CBG; and 3 = Vehicle (placebo).

Since CBG demonstrated antioxidant, anti-inflammatory, and skin health-boosting activity *in vitro*, we sought to determine whether it could provide similar benefits when applied topically to human skin. CBG was first evaluated *in vitro* to determine skin and eye irritation potential, utilizing the 3D human skin equivalents EpiDerm™ and EpiOcular™. Results showed CBG was non-irritating in both models up to 3%, which was the highest dose tested (Figure S2). Therefore, we performed a proof-of-concept inflammation clinical study via a third-party contract research organization (Princeton Consumer Research). This single-blind clinical study was performed in 20 healthy male and female volunteers with SLS-induced contact dermatitis to investigate the skin protectant and anti-inflammatory properties of 0.1% CBG serum versus placebo. SLS-induced contact dermatitis is a clinical model often used to test the anti-redness and anti-inflammatory potential of a test article and/or topical formulation [35]. CBG was formulated at 0.1% in a serum and was applied along with placebo to each subject at two distinct sites on the forearm almost daily for 15 days. Moreover, a third distinct site on the forearm was left untreated as an additional control. Skin barrier function was measured via TEWL using a Tewameter^®^ and inflammation and redness was graded visually, as described in Table 3. Results show CBG-treated sites produced statistically significantly lower TEWL values compared to untreated and placebo sites, demonstrating an increase in skin barrier function (Figure 3). Inflammation and redness induction by SLS result in moderate erythema, papules, or deep fissures with moderate-to-severe erythema in the cracks, which correlates to grade 2 on the skin irritation scale (Table 3). Application of 0.1% CBG serum after just 48 h showed a significant reduction of inflammation and redness better than placebo and untreated test sites (Figure 4). Furthermore, after 2 weeks of application, 0.1% CBG serum continued to significantly outperform the placebo, returning the skin condition almost back to baseline levels of grade 0 on the visual scale, showing either barely perceptible erythema or none (Figure 4). Altogether, these results indicate that CBG, when topically applied, clinically reduces skin inflammation and the appearance of redness and improves barrier function. Vehicle (placebo)-treated sites provided a small but significant effect in reducing TEWL and visual grade endpoints compared to untreated skin. This could be due to the presence of glycerin in the base vehicle acting as skin moisturizer.

## 3. Discussion

We demonstrate for the first time that minor cannabinoid CBG, when applied topically, clinically promotes skin health by reducing the appearance of redness and improving barrier function better than a placebo. Based on the data presented here, CBG is an attractive new candidate for dermatological use, outperforming its more well-known derivative, CBD, in several *in vitro* studies. These initial findings for CBG provide further compelling evidence for the use of cannabinoids as topical agents with multiple skin benefits and highlights key similarities and differences in activity between CBG and CBD. For instance, a topical study applying a 1% CBD solution daily to hairless mice for 14 days was shown to improve skin moisturization [36]. The authors demonstrate that CBD upregulates aquaporin-3 (AQP3), the most abundant aquaporin found in skin shown to play a key role in water and glycerol transport, skin hydration, elasticity and barrier repair [37]. However, when TEWL measurements were taken, the 1% CBD solution did not have any significant effects. These results are a sharp contrast with the findings we report here that, albeit in human and not mouse skin, 0.1% CBG serum significantly reduces TEWL (Figure 3), which suggests it may be better than CBD in maintaining hydrated skin and improving barrier function. CBD, CBG, and several other minor cannabinoids have exhibited the ability to scavenge free radicals and prevent oxidative stress [10]. While the examined cannabinoids all demonstrate antioxidant properties, differences are observed for each specific molecule. For example, utilizing the ABTS (2,2′-Azino-bis (3-Ethylbenzothiazoline-6-Sulfonic Acid)) cell-free antioxidant assay, Dawidowicz et al. showed that CBG is slightly more potent than CBD, similar to what we report here using the DPPH assay (Table 2), and that both are significantly better than another minor cannabinoid cannabinol (CBN) [10]. Additional studies examining the photoprotection potential of cannabinoids in human keratinocyte cell line (HaCaTs) and HDFs exposed to UVB and UVA, respectively, showed CBD to be the most effective protectant against UVA (although not significantly better than CBG), while CBN was the best at protecting against UVB and was significantly better than CBD and CBG [38]. The cell-based antioxidant results reported here (Table 2) support the growing literature that CBG and CBD are both potent antioxidants with a similar *in vitro* activity range. 

In recent years, the association between oxidative damage and inflammatory response have been studied in detail, showing that oxidative stress can provide a significant contribution to inflammatory response [24]. Inflammatory processes induce oxidative stress, reducing cellular antioxidant capacity. This then causes an overproduction of free radicals that react with cell membrane fatty acids and proteins, impairing their function. In addition, free radicals can lead to mutation, DNA damage and premature aging. To counter the negative effects of inflammation and oxidative stress, both types of antioxidants, endogenous (products of the body’s metabolism) and exogenous (from diet or supplements), could inhibit and prevent damage resulting from ROS as a third line of defense via removing oxidatively modified proteins and prevent the accumulation of oxidized proteins by action of proteolytic enzymes (proteinases, proteases, and peptidases) acting as “free radical scavengers” and boosting the immune defense. Therefore, in addition to causing oxidative stress, chronic exposure to UV can also accelerate skin aging. As mentioned above, cannabinoids provide photoprotection against UV-induced cell death, and CBD has also been shown to reduce the pro-inflammatory action of UVB in psoriatic keratinocytes [39] and reduce TPA-induced erythema in mouse skin [40]. Moreover, CBD, CBG, and other cannabinoids demonstrate anti-inflammatory activity in skin cells versus several different inducers [2]. We demonstrate here that CBG and CBD inhibit pro-inflammatory cytokine release from cells induced with UVB, UVA, TPA, and *C. acnes* (Table 2, Figure 2). Interestingly, in three of these four *in vitro* inflammatory assays, CBG was more potent than CBD. CBG’s effective *in vitro* anti-inflammatory profile and clinical effectiveness in reducing SLS-induced redness when applied to human skin suggests it can be an effective ingredient to use in several inflammatory skin conditions. One such condition is inflammatory acne. Clinical trials testing for anti-acne activity have previously been reported for CBD [6] and a *C. sativa* extract [5]. CBD has been shown to be an effective sebostatic agent with anti-inflammatory properties in studies utilizing sebocytes [41]. Additionally, both CBG and CBD have been shown to possess strong antibacterial activity against *C. acnes* and several other harmful skin bacteria such as *Staphylococcus aureus* (*S. aureus*) and methicillin-resistant *S. aureus* (MRSA) [42]. Given this broad activity profile, we hypothesize that CBD, CBG, and other cannabinoids could be effective at treating acne prone skin. Demonstrated here, CBG’s increased potency over CBD in reducing *C. acnes*-induced inflammation positions it as an attractive candidate as either a stand-alone treatment for acne or in combination with other cannabinoids. Moreover, *S. aureus* is a key factor in the pathogenesis of atopic dermatitis [43], thus raising the possibility that CBG may also be effective for this dermatological condition. 

While CBD and CBG modulate many targets at the gene level, the data presented here demonstrate that CBG has greater potency in modulating specific cutaneous targets. One potential explanation for these differences in activity could be the affinity CBD and CBG have for cannabinoid receptors. The endocannabinoid system (ECS) is an evolutionarily conserved signaling network that plays a key role in skin homeostasis [3]. Cannabinoid receptors 1 and 2 (CB1 and CB2) are found throughout the body, with CB1 mostly found in the central nervous system and CB2 in the peripheral nervous system. However, both CB1 and CB2 are also expressed in several different skin cell types including epidermal keratinocytes, melanocytes, hair follicles, nerve fibers, and sweat glands [44,45,46,47]. CBG has previously been reported to act as a partial agonist for both CB1 and CB2 [48], while CBD does not bind to CB2, but may affect CB1 receptor activity via an indirect method [49]. Thus, we can speculate that CBG’s ability to modulate both cannabinoid receptors may lead to its improved activity and efficacy in skin. Nevertheless, this only explains in part the potential mechanism of action for CBG, CBD, and other cannabinoids when applied topically. For example, HDFs express low levels of CB1 and CB2 only after inflammatory induction or cancer types; however, this study and others have shown that CBG and CBD effectively inhibit inflammatory and oxidative stress signaling in HDFs [50,51]. This suggests the activity observed for cannabinoids in skin could be via modulation of other receptor signaling pathways as well. One such potential target is peroxisome proliferator-activated receptors (PPARs), which have been shown to be important in regulating skin physiology and dermatological diseases [52]. Dermal fibroblasts treated with CBD activate PPARγ and decrease nuclear factor kappa B (NF-kB) levels [53]. *In silico* studies identified CBG as a PPARα/γ dual agonist [54], and a quinone derivative of CBG has also been suggested to modulate PPARγ [55]. Future research could examine if CBG activates PPARγ and PPARα in dermal cells given their key function in epidermal biology. Another possible dermal target is the G-protein-coupled A2a adenosine receptor, which is expressed in human sebocytes. Olah et al. showed that sebocytes treated with CBD conferred anti-inflammatory activity through the A2a receptor-dependent upregulation of tribbles homolog 3 (TRIB3) leading to inhibition of the NF-κB signaling pathway. The authors hypothesized that the A2a receptor was likely the main target for the anti-inflammatory activity of CBD in human sebocytes [41]. Moreover, CBG has previously been reported to activate transient receptor potential ankyrin 1 (TRPA1), a key pain and inflammation target [56], and to confer anti-inflammatory effects via the modulation of transient receptor potential vanilloid-3 and 4 (TRPV-3 and TRPV-4) [57]. As reported here, CBG and CBD both successfully inhibit *C. acnes*-TLR-2-induced inflammation in keratinocytes (Figure 2b), suggesting a possible role of cannabinoids in modulating TLR signaling, which is also an emerging dermatological target [31]. This is further supported by reports of CB1 being critical for the innate immune response of TLR-4 [58] and cannabinoids shown to modulate TLR-3 signaling [59]. Thus, while additional research remains to be performed to better characterize the activity of CBG in skin, the early picture suggests that its pleiotropic effects are conferred through both cannabinoid receptors as well as several other receptors in the skin.

## 4. Materials and Methods

### 4.1. Reagents and Chemicals

All reagents were purchased from Sigma Chemical Co. (St. Louis, MO, USA). Cannabidiol was purchased from Mile High Labs (Broomfield, CO, USA). Organic solvents were purchased from Fisher Scientific (Hampton, NH, USA). 

### 4.2. Cannabigerol Production

CBG was prepared via biosynthesis using yeast strain CEN.PK2-1D engineered with an integrated pathway of genes encoding the four enzymes (acyl-activating enzyme; tetraketide synthase; olivetolic acid cyclase; and prenyltransferase) that convert hexanoic acid to cannabigerolic acid (CBGA). The CBGA biosynthesis product was decarboxylated to CBG by heating in the presence of a weak base. The resulting CBG material is a slightly off-white powder with >99% purity. Samples are available by contacting Willow Biosciences, Inc. (Calgary, AB, Canada).

### 4.3. Cell Culture

Primary normal human epidermal keratinocytes (NHEKs) and human dermal fibroblasts (HDFs) were purchased from ThermoFisher (Carlsbad, CA, USA). NHEKs were cultured in EpiLife™ medium supplemented with Human Keratinocyte Growth Supplement (HKGS). HDFs were cultured in Dulbecco’s modified Eagle’s medium (DMEM) supplemented with 10% fetal bovine serum (FBS). Both cell types were seeded with growth factor-supplemented medium for 24 h and later, the medium was replaced with supplement-depleted media for an additional 24 h before treatments. Three-dimensional tissue models EpiDerm™, EpiDerm-FT™, and EpiOcular™, were purchased from MatTek Corp. (Ashland, MA, USA) and acclimated for 1–24 h before topical treatments. Cells and tissues were incubated at standard conditions (5% CO_2_; 37 °C).

### 4.4. Gene Microarray

Differential gene expression analyses were conducted using full-thickness 3D skin equivalents containing epidermal keratinocytes and dermal fibroblasts (EpiDerm-FT™, MatTek Corp., Ashland, MA, USA). The vehicle used was ethoxydiglycol (Sigma Chemical Co., St. Louis, MO, USA). Treatments (5 mg/mL, 0.5% *w*/*v*) were applied to the surface of the skin cultures for 24 h in triplicate. Total RNA was isolated from the skin cultures, and global gene expression profiling was analyzed by Affymetrix Human Clariom™ S arrays and data visualized using Transcriptome Analysis Console (TAC) Software (ThermoFisher Scientific, Wilmington, DE, USA). Sample preparation, microarray hybridization, scanning and quality control were carried out at Advanced Biomedical Labs (Cinnaminson, NJ, USA). Significant expression changes between the treated and control group (vehicle-only) were filtered using fold change ≥2 (up-regulated) or ≤2 (down-regulated) and *p* value < 0.05 using empirical Bayes ANOVA. Significant up- and down-regulated genes were analyzed for gene ontology (GO) terms using Metascape [60] website (http://metascape.org/gp/index.html#/main/step1 (accessed on 10 February 2021)). 

### 4.5. Antioxidant Assays

Cell-free antioxidant capacity assay was performed using the 2,2-diphenyl-1-picrylhydrazyl (DPPH) antioxidant assay obtained from Cayman Chemical Company (Ann Arbor, MI, USA). The assay determined the overall antioxidant capacity of the test materials by the ability to reduce DPPH to diphenylpicrylhydrazine (DPPHH), which involved the suppression of optical density (OD) at 530 nm using a plate reader (Envision-PerkinElmer; Waltham, MA, USA). Cell-based antioxidant assay was performed using HDFs seeded into black-wall 96-well plates. Cells were pre-treated for 3 h with and without test compounds and then labeled with 50 µM of dichloro-dihydro-fluorescein diacetate (DCFH-DA) for 1 h. Later, DCFH-DA was removed, intracellular ROS was induced with 0.1 mM H_2_O_2_ for 20 min, and total fluorescence (Excitation = 485 nm; Emission = 535 nm) was measured using an Envision plate reader (PerkinElmer, Waltham, MA, USA). Ascorbic acid was used in both antioxidant assays as positive control.

### 4.6. Bacteria Culture

*Cutibacterium acnes* (*C. acnes*) (ATCC^®^ 6919™; Manassas, VA, USA) was cultured under anaerobic conditions at 37 °C for 72 h using Reinforced Clostridial Medium (RCM) and BD GasPak™ system (BD Biosciences, San Jose, CA, USA).

### 4.7. Anti-Inflammatory Assays

Cells were pre-incubated for 2 h with test materials (1% *v*/*v* ethanol vehicle) in fresh supplement-depleted media in triplicate. NHEKs were induced by 5 ng/mL 12-O-tetradecanoyl-phorbol-13-acetate (TPA) or live *C. acnes* (10^7^ CFU/mL) for 24 h or irradiated with 25 mJ/cm^2^ broadband 305–12 nm ultraviolet B (UVB) (Daavlin; Bryan, OH, USA) without test materials and incubated for an additional 18 h. HDFs were irradiated with 12.5 J/cm^2^ ultraviolet A (UVA) (350–12 nm) and later incubated without test materials for an additional 18 h. Media supernatants were harvested after induction and used to measure levels of Interleukin-1β (IL-1β), Interleukin-6 (IL-6), Interleukin-8 (IL-8), collagenase (pro-MMP1) or tumor necrosis factor-α (TNF-α) by sandwich ELISA kits (BD Biosciences, San Jose, CA, USA; R&D Systems, Minneapolis, MN, USA). Cells were subject to viability tests by MTS [3-(4,5-dimethylthiazol-2-yl)-5-(3-carboxymethoxyphenyl)-2-(4-sulfophenyl)-2H-tetrazolium, inner salt] reduction assay (Promega; Madison, WI, USA) to determine the maximum non-toxic concentrations of each material (Figure S1). 

### 4.8. Skin and Eye Irritation Assays

Irritation assays were conducted using EpiDerm™ and EpiOcular™ tissue models (MatTek Corp., Ashland, MA, USA). Tissues were acclimated and then treated topically with CBG (≤30 mg/mL; 3% *w*/*v*) formulated in Captex^®^ 355 (Abitec Corp., Columbus, OH, USA) or positive control irritants SDS (5% *w*/*v*) or methyl acetate. Tissue viability levels were measured by MTT (3-(4,5-dimethylthiazol-2-yl)-2,5-diphenyltetrazolium bromide) reduction assay. The levels of tissue viability after each treatment were compared to the vehicle-treated group to estimate the potential for skin or ocular irritation. 

### 4.9. Clinical Study

A single-blind clinical study in 20 healthy male and female volunteers was conducted at Princeton Consumer Research (Study ID and protocol#: WILIFL1M; Essex, UK) to investigate the skin protectant and anti-inflammatory properties of 0.1% CBG serum versus placebo on chemical-induced damaged skin. Written informed consent conforming to the International Council for Harmonisation (ICH) E6(r2) was obtained from each subject. Inflammation induction was performed at baseline (day 0) by applying 0.75% sodium lauryl sulfate in water (SLS) to adhesive occlusive patches and affixing them to three sites on the volar forearm for 24 h. Test articles were applied to the same two sites, leaving the third site untreated, on days 1, 2, 3, 4, 7, 8, 9, 10, 11, 14 and 15 of the study. For each application, test materials were applied and allowed to sit on the surface of the treatment site for approximately 10 min. After 10 min exposure, treatment was removed, measurements taken, and then treatments were reapplied with subjects instructed to not remove the bandage until the next study day. Transepidermal Water Loss (TEWL) measurements were taken using the Tewameter^®^ TM300 instrument (Courage and Khazaka, Germany), and readings were taken on day 0 (before SLS patch application), day 1 (after SLS patch removal), and on all treatment days approximately 10 min after test article application. On days 4, 9, and 15, additional TEWL readings were taken prior to treatment reapplication. TEWL readings taken on day 0 before application of the SLS patches served as baseline readings. Clinical grading for the skin irritation (Table 1) of test sites was performed on day 0 (before SLS patch application), after SLS patch removal on day 1, 10 min after first application on day 1, and post-test article removal on days 1, 2, 3, 4, 7, 8, 9, 10, 11 and 14. The application site was photographed using a point and shoot camera at baseline, day 1, 7 and 14.

### 4.10. Statistical Analysis

Statistical significance was determined by ANOVA followed by a Dunnett multiple comparisons test using *p*-values less than 0.05 as a significant difference. For all *in vitro* assays, samples were run in triplicate. Cytokine dose–response curves were generated by fitting data with the Hill, three-parameter equation using the Sigma Plot software (Palo Alto, CA, USA), from which the inhibitory concentration at 50% (IC_50_) values and maximum inhibition were determined.

## 5. Conclusions

We demonstrate here for the first time that CBG in a clinical study is both safe and effective in promoting skin health by reducing the appearance of redness and improving barrier function better than a placebo. CBG exhibits a broad range of *in vitro* activity, including antioxidant, anti-inflammatory, anti-acne, and anti-aging properties. Based on the data reported here, CBG is an attractive new candidate for dermatological use, outperforming its more well-known derivative, CBD. Lastly, utilizing our novel yeast fermentation technology platform, we can produce CBG and other minor cannabinoids with higher purity using a more sustainable and cost-effective process compared to *C. sativa* plant extraction and chemical synthesis.

## Figures and Tables

**Table 1:** Summary of antioxidant activity.

Compound	IC_50_^1^	
	Cell-Free Assay (DPPH)	Cell-Based Assay (DCFH-DA)
CBD	286 µg/mL (910 µM)	0.001 ng/mL (0.003 nM)
CBG	159 µg/mL (502 µM)	0.001 ng/mL (0.003 nM)
Ascorbic Acid	5 µg/mL (502 µM)	>100 ng/mL (5.6 nM)

^1^ IC_50_ = Inhibitory concentration at 50%. Results represent average cumulative data from 3 independent experiments. IC_50_ values were determined from dose–response curves using a four-parameter logistic curve fit.

**Table 2:** Summary of *in vitro* photoaging activity.

Compound	IC_50_^1^	
	HDFs-UVA (IL-6)	NHEKs-UVB (TNFα)
CBD	>0.1 µg/mL (0.3 µM)	0.009 µg/mL (29.8 nM)
CBG	0.1 µg/mL (0.3 µM)	0.005 µg/mL (14.7 nM)
Clobetasol	ND	>1 µg/mL (2142 nM)
Ascorbic Acid	>1 µg/mL (5.7 µM)	ND

^1^ IC_50_ = Inhibitory concentration at 50%. Results represent average cumulative data from 3 independent experiments. IC_50_ values were determined from dose–response curves using a four-parameter logistic curve fit. ND = not determined.

**Table 3:** Grading scale for skin irritation in clinical study.

Grading Scale	Observation on Treated Site
0.0	No apparent cutaneous involvement
0.5	Faint, barely perceptible erythema, or slight dryness (glazed appearance)
1.0	Faint but definite erythema, no eruptions or broken skin or no erythema but definite dryness; may have epidermal fissuring
1.5	Well-defined erythema or faint erythema with definite dryness, may have epidermal fissuring
2.0	Moderate erythema, may have a very few papules or deep fissures, moderate-to-severe erythema in the cracks
2.5	Moderate erythema with barely perceptible edema or severe erythema not involving a significant portion of the patch (halo effect around the edges), may have a few papules or moderate-to-severe erythema
3.0	Severe erythema (beet redness), may have generalized papules or moderate-to-severe erythema with slight edema (edges well defined by raising)
3.5	Moderate-to-severe erythema with moderate edema (confined to patch area) or moderate-to-severe erythema with isolated eschar formations or vesicles
4.0	Generalized vesicles or eschar formations or moderate-to-severe erythema and/or edema extending beyond the area of the patch

## Data Availability

Data available upon request to corresponding author: c.savile@willowbio.com.

## Supplementary Materials

Figure S1: Cell viability after test material treatments, Figure S2: CBG is safe and non-irritating when applied to 3D skin and eye models in vitro, Gene Microarray Data vs. Vehicle.

## Sample Availability

Samples of the compounds are available from the corresponding author: c.savile@willowbio.com.
