# Supplementary material for: In Vitro and Clinical Evaluation of Cannabigerol (CBG) Produced via Yeast Biosynthesis: A Cannabinoid with a Broad Range of Anti-Inflammatory and Skin Health-Boosting Properties
Source: Molecules. 2022 Jan 13;27(2):491. doi: 10.3390/molecules27020491 (PMC8778347; doi:10.3390/molecules27020491)
Supplement: Supplementary file 1 [file molecules-27-00491-s001.zip › molecules-1541828-supplementary.pdf]

Supplemental Figures

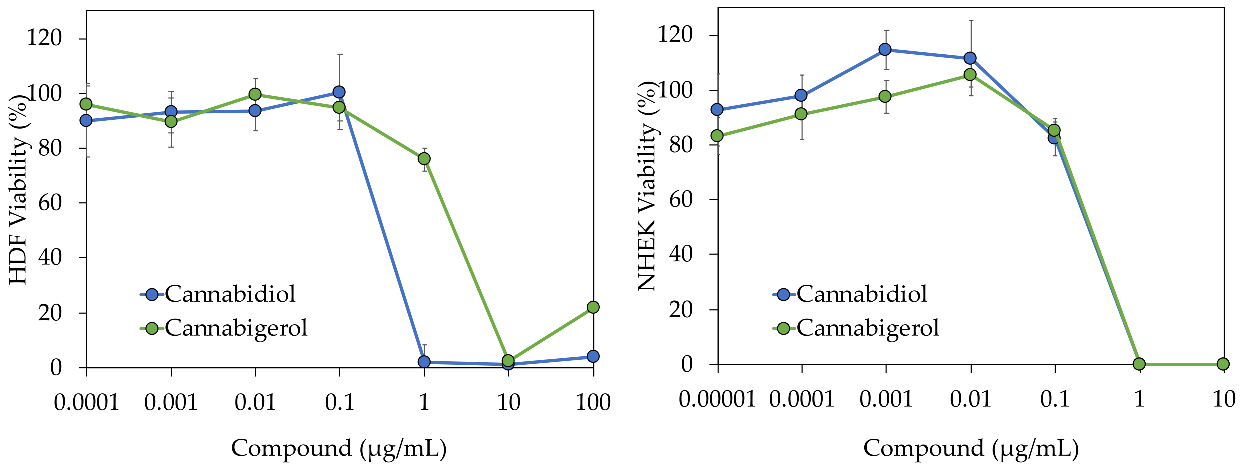

(a) **(b)**

Figure S1. Cell viability after test material treatments for 24 hours. (**a**) Human dermal fibroblasts (HDFs) and (**b**) Normal human epidermal keratinocytes (NHEKs). Data represent mean ± SEM.

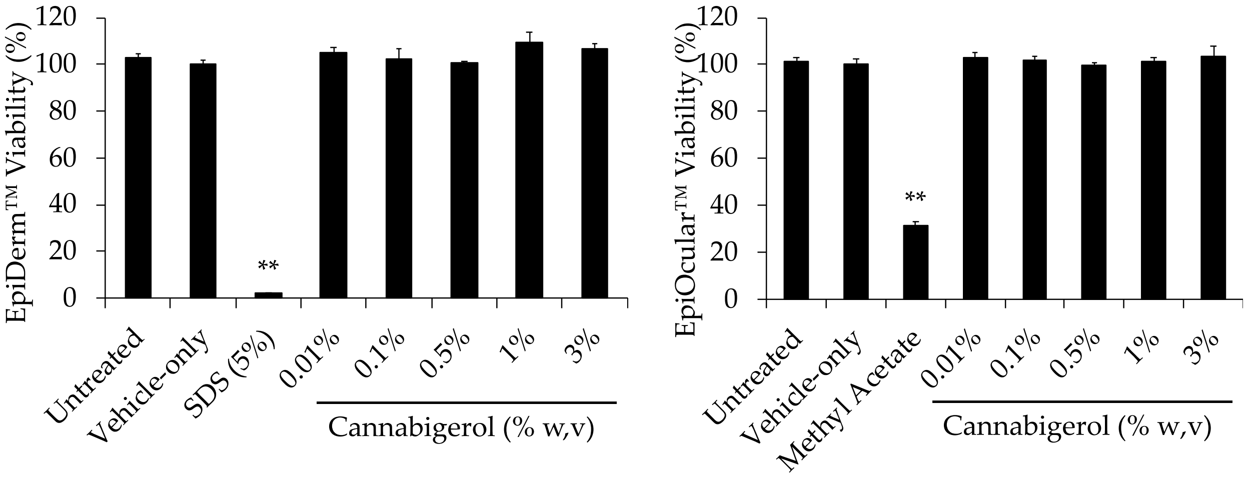

Figure S2. CBG is safe and non-irritating when applied to 3D skin and eye models *in vitro*. CBG was tested for skin and eye irritation in 3D reconstructed human tissue models. (A) EpiDerm™ and (B) EpiOcular™ (MatTek®) tissues were acclimated for 1-24 hours and then treated topically with CBG formulations (≤3%) or positive controls SDS (5%) or methyl acetate. Tissue viability levels were measured by MTT reduction assay. The levels of tissue viability after each treatment were compared to vehicle group to estimate the potential for skin or ocular irritation. **$p \leq 0.01$ compared with untreated tissues.
